# Indications and Outcomes of the Components Separation Technique in the Repair of Complex Abdominal Wall Hernias: Experience From the Cambridge Plastic Surgery Department

**Published:** 2013-09-16

**Authors:** Shola Adekunle, Nicholas M. Pantelides, Nigel R. Hall, Raaj Praseedom, Charles M. Malata

**Affiliations:** ^a^Departments of Plastic and Reconstructive surgery, Addenbrooke's University Hospital, Cambridge, UK; ^b^Departments of General Surgery, Addenbrooke's University Hospital, Cambridge, UK

**Keywords:** abdominoplasty, components separation, incisional hernias, laparotomy wound dehiscence, mesh inlay/onlay

## Abstract

**Objectives:** The components separation technique (CST) is a widely described abdominal wall reconstructive technique. There have, however, been no UK reports of its use, prompting the present review. **Methods:** Between 2008 and 2012, 13 patients who underwent this procedure by a single plastic surgeon (C.M.M.) were retrospectively evaluated. The indications, operative details, and clinical outcomes were recorded. **Results:** There were 7 women and 6 men in the series with a mean age of 53 years (range: 30-80). Patients were referred from a variety of specialties, often as a last resort. The commonest indication for CST was herniation following abdominal surgery. All operations except 1 were jointly performed with general surgeons (for bowel resection, stoma reversal, and hernia dissection). The operations lasted a mean of 5 hours (range: 3-8 hours). There were no major intra- and postoperative problems, except in 1 patient who developed intra-abdominal compartment syndrome, secondary to massive hemorrhage. All patients were satisfied with the cosmetic improvement in their abdominal contours. None of the patients have developed a clinical recurrence after a mean follow-up of 16 months (range: 3-38 months). **Conclusions:** The components separation technique is an effective method of treating large recalcitrant hernias but appears to be underutilized in the United Kingdom. The management of large abdominal wall defects requires a multidisciplinary approach, with input across a variety of specialities. Liaison with plastic surgery teams should be encouraged at an early stage and the CST should be more widely considered when presented with seemingly intractable abdominal wall defects.

Large and complex defects of the anterior abdominal wall occur most commonly due to herniation following abdominal surgery, but may also be caused by trauma, infection, or tumour resection. Apart from the obvious aesthetic abnormality, they can also lead to physical symptoms and poor protection of the intra-abdominal viscera. The management of such defects poses a surgical challenge, particularly since primary repair has reported recurrence rates as high as 50%.^1^ The use of synthetic mesh decreases recurrence rates by up to 20%,^2^ but this carries the risk of mesh infection, exposure, and extrusion. It is also contraindicated in the presence of gross contamination or infection.

The components separation technique (CST), first described by Ramirez et al,^3^ provides tensionless closure of large, full thickness anterior abdominal wall defects with autologous tissue. It was originally described without the use of prosthetic material. Quoted recurrence rates range from 0% to 30%.^1^^,^^4^^,^^5^ Subsequent modifications of the CST have incorporated the use of prosthetic materials, with consequent reductions in hernia recurrence rates.^6^

Despite the versatility of the CST and its low recurrence rates, the technique does not appear popular within the United Kingdom and, to our knowledge, this is the first published series from a UK hospital. Patients were referred to the plastic surgery service from a variety of specialties, often as a last resort. We present our experience with the CST and report surgical outcomes and morbidity, with the aim to raise awareness of this technique among various specialties and to promote early liaison with plastic surgical teams in the management of these complex cases.

## PATIENTS AND METHODS

A retrospective review of patients who underwent CST for repair of ventral abdominal wall hernias by a plastic surgeon (C.M.M.) between 2008 and 2012 was performed. Demographic data, coexisting medical problems, indications for surgery, referring speciality, previous repairs, length of surgery, use or otherwise of mesh, and surgical outcomes were collected (Table 1). Follow-up was measured from the date of surgery to the most recent outpatient clinic (office) visit.

### Operative technique

**Figure 1:**
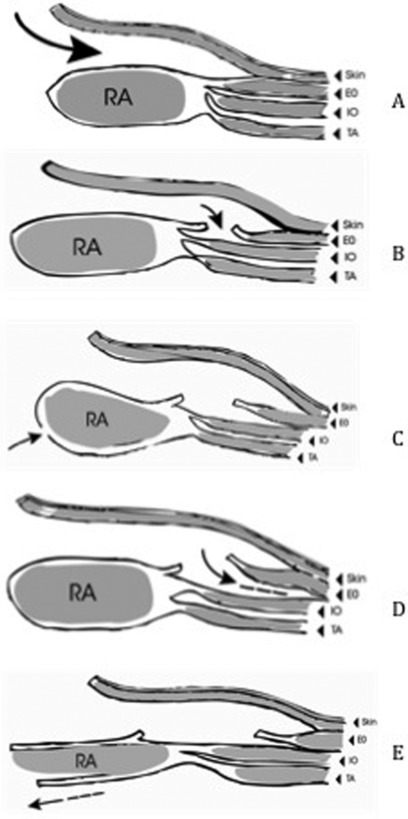
Intraoperative technique. (*a*) Using coagulation diathermy, the skin and subcutaneous tissue are dissected free from the anterior rectus sheath and the external oblique (EO) aponeurosis, extending laterally as far as the anterior-superior iliac spine. (*b*) The EO aponeurosis is incised longitudinally about 2 cm lateral to the rectus abdominis (RA) muscle. (*c*) The EO aponeurosis and EO muscle are separated from the underlying internal oblique (IO) muscle as far laterally as possible, up to the mid-axillary line. (*d*, *e*) The posterior rectus sheath is then incised longitudinally and separated from the RA muscle to provide further medial advancement of the RA-IO myofascial complex.

All patients had general anesthesia and were given broad-spectrum antibiotics at induction. Routine thromboprophylaxis with compression stockings and low-molecular-weight heparin was administered to all patients. With patients in the supine position, access to the abdomen was obtained via a midline incision, usually performed by the general surgeons to divide any bowel adhesions to the scar or skin graft. In the presence of a widened scar or skin graft covering the intestines, this was resected. The intra-abdominal viscera were then carefully freed from the ventral abdominal wall after which the plastic surgeons performed the components separation closure of the abdomen in the standard fashion (Fig 1).

Midline closure of the abdomen was performed with running looped “0” nylon (± interrupted “0” ethibond) in 2 layers. Where mesh reinforcement was deemed necessary, this was used as an onlay, secured with continuous 3’0 Prolene to the lateral edge of the external oblique aponeurosis.

### Postoperative care

Suction drains remained in situ until the output was less than 30 to 50 mL in 24 hours. The patients were instructed to wear a supportive binder and avoid heavy lifting and other strenuous activities for at least 3 months.

## RESULTS

From April 2008 to March 2012, 13 patients who underwent components separation to repair ventral abdominal wall hernias/defects were identified. There were 7 women and 6 men. Their mean average age was 53 years (range: 30-80) with a mean follow-up of 16 months (range: 3-38).

The most common indication for CST was previous abdominal surgery, in 9 of the 13 patients (Table 1). Unusual indications included polyhydramnios and multiple close pregnancies in one patient, and exercise-induced herniation in another. Three patients had had previous attempts at repair (one of these due to scarring from 21 previous abdominal procedures).

The average length of surgery was 5 hours (range: 3-8). All patients had bilateral division of the external oblique aponeurosis; the posterior sheath was also incised free if present. Seven of the 13 patients had reinforcement of their repairs with an onlay Prolene mesh.

A major postoperative complication occurred in 1 patient, who developed an intra-abdominal compartment syndrome secondary to massive postoperative hemorrhage, following concurrent excision of multiple peritoneal/mesenteric calcifications. Three patients developed seromas, requiring drainage and 1 patient had a minor wound breakdown, which required debridement and suturing under general anesthetic 7 weeks postoperatively.

**Figure 2:**
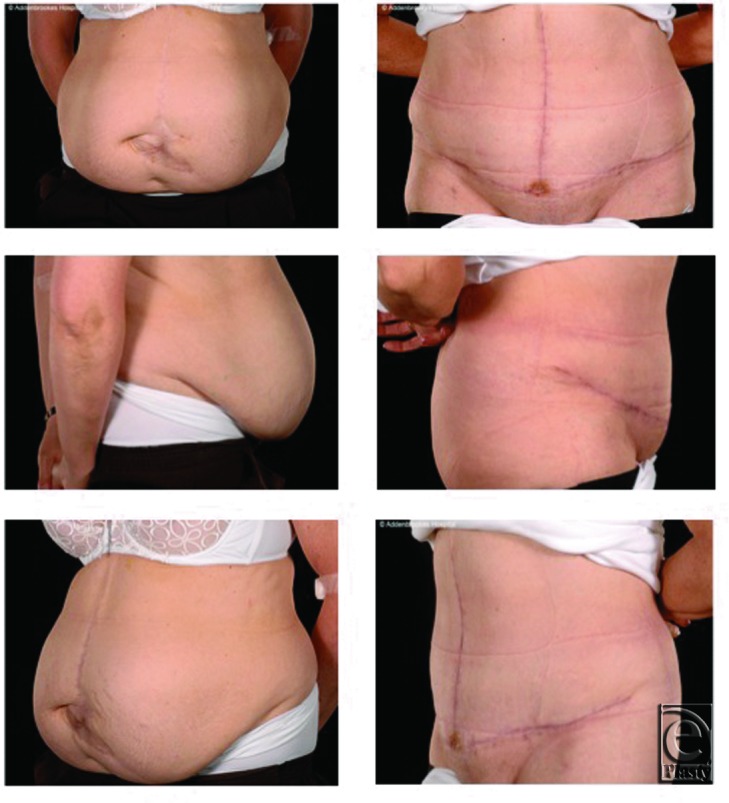
Pre- and postoperative appearances. (*Left*) A 62-year-old woman who developed a central abdominal hernia following a hemicolectomy for diverticular disease (patient 5). (*Right*) Postoperative appearance 3 months after components separation technique, demonstrating a much improved abdominal contour.

**Figure 3:**
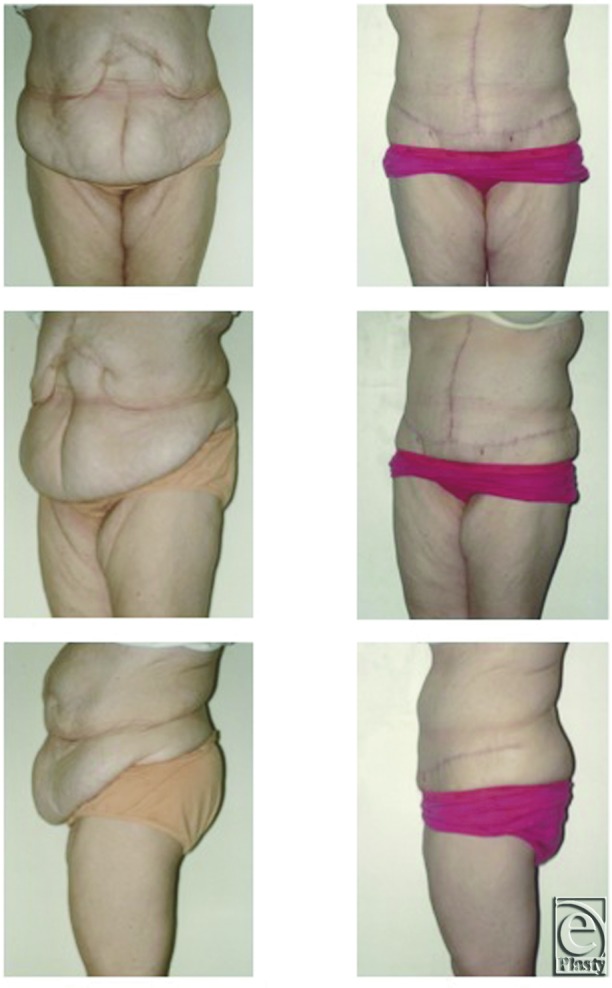
Pre- and postoperative appearances. (*Left*) A 35-year-old woman with a large upper abdominal incisional hernia following previous abdominal surgeries for pancreatectomy and gastric bypass surgery (patient 10). She had 5 previous attempts at hernia repair prior to referral and underwent components separation technique repair and scar revision via a Fleur-de-Lys abdominoplasty. (*Right*) Postoperative appearances at 3 month showing excellent abdominal contour and an absence of herniation.

All patients were satisfied with the cosmetic improvement in their abdominal contours (Figs 2 and 3). None of the patients have developed a clinical recurrence after a mean follow of 16 months.

## DISCUSSION

Complex abdominal wall hernias/defects remain a challenging problem for reconstructive surgeons. Apart from the cosmetic disfigurement and physical symptoms, anterior abdominal wall defects result in loss of domain and poor protection of the internal organs. Despite the high incidence of defects unsuitable for direct closure, there is little consensus as to the optimum technique for repair. Options include the use of prosthetic mesh, fascia lata or dermal grafts, pedicled or free tissue transfer, and the CST.^7^

The CST enables significant advancement of the rectus abdominis/internal oblique/transversus abdominis muscles toward the midline, with preservation of the neurovascular bundles, resulting in a dynamic longstanding repair. Data from the only prospective, randomized-controlled trial to compare autologous CST hernia repair with prosthetic mesh hernia repair showed a favorable outcome for the CST, given the frequency of wound complications and subsequent prosthetic loss in the mesh group.^8^

As the CST was first described over 2 decades ago, data are now available from large case series and with long-term follow-up, demonstrating the efficacy of this technique. For instance Ko et al^6^ reviewed 200 patients who underwent CST and showed a 22.8% hernia recurrence rate for primary CST after a mean follow-up period of 10 months. A separate study demonstrated a 19.8% hernia recurrence rate at a mean follow-up of 4.4 years.^9^

However, while the CST is commonly performed overseas, it does not appear to be a popular technique in the United Kingdom, with no published series from British hospitals specifically addressing this subject. In addition, it is a technique that is at the interface of a number of surgical specialties. Our series demonstrates that it is a safe and effective technique, even when performed by a relatively low-volume operator, with no hernia recurrences to date. In addition, because it utilizes local, innervated tissue, it maintains the abdominal contour and provides an acceptable cosmetic outcome (Figs 2 and 3).

Wound complications such as dehiscence, infection, hematoma, and seroma have been reported in several series and are thought to relate to the undermining of the subcutaneous tissues, transection of epigastric perforating vessels, and creation of dead space during the CST.^4^^,^^5^ In our series, 3 patients developed a seroma requiring repeated percutaneous drainage and another developed a minor wound breakdown (Table 1). We consider this wound morbidity to be acceptable given the complex nature of the abdominal defects in the series. Kingsnorth et al^10^ have suggested that the application of fibrin sealant where skin flaps are dissected past the linea semilunaris may help to reduce the incidence of seroma. In addition, the recent introduction of endoscopic and minimally invasive techniques have been shown to reduce wound complications, possibly by preserving local blood supply and thereby minimizing tissue ischemia.^11^ However, with the large defects that our patients presented with in this series, endoscopic techniques are of limited value.

The CST originally described by Ramirez et al was used to bridge the fascial gap without the use of prosthetic material. However, modifications to this technique have incorporated a prosthetic mesh to augment the repair, and this has been shown to lower hernia recurrence, when compared to CST alone.^6^ Seven of the 13 patients in our series required mesh (Prolene) reinforcement of the CST to reduce tension on the midline closure. One patient who developed compartment syndrome postoperatively eventually required a Permacol mesh to enable temporary closure of the abdomen. While concerns remain regarding the risk of infection or extrusion of the mesh, the Ko series did not demonstrate a significant increase in major or minor postoperative complications when a mesh was used.^6^

The patients in our series were referred from a wide variety of specialities, including general surgeons, other surgical specialities, and primary care physicians. Given the complex nature of these cases, and the interaction with multiple specialities, it is important to undertake these procedures in a multidisciplinary fashion and, where possible, patients should be assessed by all teams involved preoperatively. All surgical specialities involved with the operation must clarify to each other what they intend to do and what they expect of the other team, in order to optimize the surgery. The majority of cases in our series were performed in conjunction with the general surgeons, and this appears to be in keeping with other centers that perform the CST.^5^^,^^12^ It is also important that joint care is continued postoperatively.

## CONCLUSION

The CST is a safe and effective technique, which can be used to treat complex abdominal hernias/defects. It requires close cooperation of multiple surgical specialities and, in defects where primary closure is unsuitable, liaison with the plastic surgical team should be considered at an early stage. The technique is associated with a low rate of hernia recurrence and an acceptable cosmetic outcome and, although it represents an important technique in trunk reconstruction, it appears to be currently underutilized (or underreported) within UK hospitals.

## Figures and Tables

**Table 1:** Patients’ clinical details

Patient	Age	Sex	Cause of hernia	Referring speciality	Comorbidity	Previous repair of hernia (number of attempts)	Length of surgery, h	Use of mesh	Follow-up (months from surgery to last clinic visit)	Complications
1	42	F	Postpartum polyhydramnios	Obstetrics and gynecology	Nil	No	7	Prolene	34	Nil
2	73	M	Laparotomy for renal cancer	Transplant	Mild asthma, Type 1 diabetes.	No	4	Prolene	24	Nil
3	60	M	Multiple abdominal operations	Upper GI	Type 2 diabetes	Yes (21)	7	Prolene	38	Recurrent Pain
4	57	M	Exercise induced diastasis	Upper GI	Nil	No	3	No mesh	27	Seroma
5	62	F	Laparotomy for diverticular disease	Colorectal	Nil	No	5	Prolene	12	Nil
6	47	M	Road traffic accident with abdominal trauma	Colorectal	Nil	No	7	Permacol	8	Abdominal compartment syndrome
7	52	F	Laparotomy for ovarian cancer	Obstetrics and gynecology	Nil	Yes (2)	6	Prolene	38	Nil
8	30	F	Laparotomy for renal transplant	Transplant	Type 1 diabetes, renal failure	No	4	No mesh	4	Nil
9	76	M	Laparotomy for ruptured abdominal aortic aneurysm	Vascular	Rectal cancer, renal failure	No	8	No mesh	3	Nil
10	35	F	Previous pancreatectomy, gastric bypass surgery, Cesarean section	Hepatobiliary	Hypothyroidism	Yes (5)	3	No mesh	6	Minor wound breakdown
11	38	F	Rectal divarication after twin pregnancy	General practitioner	Mild asthma	No	5	Prolene	4	Seroma
12	80	M	Multiple abdominal operations	Colorectal	Type 2 diabetes, hypertension, Ischaemic Heart Disease, asthma, diverticulosis	No	5	Prolene	3	Seroma
13	40	F	Cesarian sections (3) and 1 twin pregnancy	General practitioner	Nil	No	4	No mesh	4	Nil
